# Characteristics and correlation analysis of spino-pelvic sagittal parameters in elderly patients with lumbar degenerative disease

**DOI:** 10.1186/s13018-019-1156-3

**Published:** 2019-05-09

**Authors:** Qiang Wang, Chang-Tai Sun

**Affiliations:** 1 Department of Emergency, The First Affiliated Hospital of Xi’an Jiaotong University, Xi’an, Shaanxi China; 2 Department of Orthopedics, Beijing Hospital, National Center of Gerontology, No.1 Dahua Road, Dong Dan, Beijing, 100730 China

**Keywords:** Elderly patients, Lumbar degenerative disease, Sagittal parameters

## Abstract

**Background:**

Retrospective analysis of the characteristics and correlation of spino-pelvic sagittal parameters in elderly patients with lumbar degenerative disease.

**Methods:**

Eighty-seven patients with lumbar degenerative disease, with an average age of 75.4 years old, were enrolled in the observation group. They were all from the orthopedics department of Beijing Hospital and got enrolled in this study from August 2015 to October 2017. Another 80 volunteers, with an average age of 74.5 years old, were enrolled in the control group. Standing lateral radiographs of the full-length spine were taken for all subjects. The following spino-pelvic sagittal parameters were measured: sagittal vertical axis (SVA), thoracic kyphosis (TK), lumbar lordosis (LL), pelvic incidence (PI), sacral slope (SS), and pelvic tilt (PT). Statistical analysis was performed to compare the differences of the parameters between groups, and the correlation analysis was also performed.

**Results:**

SVA, PI, and PT of the observation group were significantly higher than those of the control group (*p* < 0.01), while LL and SS were significantly lower in the observation group (*p* < 0.01). No significant differences were found in TK between the two groups. The correlation analyses showed that PI was significantly correlated with SS and PT in both the observation group (*p* < 0.01) and the control group (*p* < 0.01), so as the SVA-PI (*p* < 0.05) and SVA-PT (*p* < 0.01). SS-PT was also significantly correlated in the observation group (*p* < 0.01) and in the control group (*p* < 0.05). LL was significantly correlated with all the other parameters in the observation and control groups, including SVA (*p* < 0.01; *p* < 0.01), TK (*p* < 0.01; *p* < 0.01), PI (*p* < 0.01; *p* < 0.01), SS (*p* < 0.01; *p* < 0.01), and PT (*p* < 0.01; *p* < 0.01). SVA-SS (*p* < 0.05), TK-PI (*p* < 0.05), and TK-SS (*p* < 0.01) were significantly correlated in the control group but not in the observation group.

**Conclusion:**

Reduced coordination of the spine and pelvis in elderly patients with lumbar degenerative disease was observed. Many of the cases were in the state of sagittal imbalance, with the trunk center of gravity moving forward, the integral sagittal alignment becoming straight, and the pelvic posterior tilt increasing. Pelvic parameters were significantly correlated with each other, which may affect the sagittal curve of the spine. LL was a core parameter that significantly correlated with various sagittal parameters.

## Introduction

Lumbar degenerative disease is commonly found in elderly patients presenting to the spine clinic in daily practice. It is a general term that comprises a group of conditions associated with aging and degenerative changes of the lumbar spine, including lumbar disc herniation, lumbar spinal stenosis, lumbar degenerative spondylolisthesis, etc. Patients often have symptoms such as low back pain, difficulty walking, and inability to continually look straight ahead when upright. Some cases of the lumbar degenerative disease progress, or get worse over time, causing chronic pain and difficulty with daily tasks.

It has been recognized that the analysis of spino-pelvic sagittal characteristics is important in optimizing the management of lumbar degenerative disease [1, 2]. The spino-pelvis sagittal parameters consist of spinal parameters and pelvic parameters. In spinal parameters, sagittal vertical axis (SVA) is widely accepted as a reliable indicator of sagittal balance [3]; thoracic kyphosis (TK) and lumbar lordosis (LL) respectively reflect the kyphosis angle and the lordosis angle of the spine. In pelvic parameters, pelvic incidence (PI) was reported as the parameter to describe the pelvic morphology, in which the value would remain constant in adulthood [4]. Sacral slope (SS) is a posture-related parameter that is influenced by the position of the pelvis. SS determines the curvature of the lower lumbar and is closely related to LL [5]. Pelvic tilt (PT) is also a posture-related parameter, which indicates the spatial orientation of the pelvis and reflects the pelvic flexion or posterior tilt angle. PT increases when the pelvis tilts backward, while the value decreases when the pelvis flexes. These pelvis orientations are important mechanisms to compensate for sagittal imbalance.

Normal values of spino-pelvic sagittal parameters vary greatly from person to person [6]. Compared to young people, sagittal parameters of elderly have their own characteristics due to degenerative deformity. Many of the studies have used healthy young adults as control groups [7–10], and the research on the normal range of sagittal parameters in elderly is lacking. To restore the sagittal balance and obtain a better treatment outcome, appropriate correction of sagittal plane alignment is essential [11, 12]. This study aims to investigate the characteristics of the spino-pelvic sagittal parameters of elderly patients with lumbar degenerative disease by comparison with age-matched control.

## Patients and methods

### Patient characteristics

The subjects were selected according to the eligibility criteria. The main inclusion criteria were (1) subjects with age ≥ 70 years old; (2) the observation group consisted of patients with lumbar degenerative disease, in which (a) patients presented low back pain and radiating pain in the lower extremity unilaterally and might have paresthesia in the lower extremity, (b) the symptoms were not relieved after > 6 months of non-surgical treatment, and (c) confirmation of diagnosis by X-ray, MRI, and/or other imaging examinations; (3) while the control group consisted of subjects with no significant long-term low back pain; (4) both groups of subjects had taken the standing lateral radiographs of the full-length spine (including bilateral femoral heads), and all the images were measurable and of high quality. The main exclusion criteria were (1) a history of trauma or surgery of the spine, pelvis, or lower extremity and (2) a history of infection, tuberculosis, or cancer of the spine. We recruited the elderly volunteers in the control group through community centers in the area and did not include patients who visited our department with a disease other than lumbar degenerative disease.

### Measurements of parameters

All of the full-length standing lateral radiographs were performed using the same DR radiator by a single radiologist who was blinded to the grouping information of the subjects. Subjects were in a natural standing position when taking the images, with eyes looking forward, fingers gently resting on the bilateral clavicle, elbow fully flexed, and hip and knee joints fully extended.

**Fig. 1:**
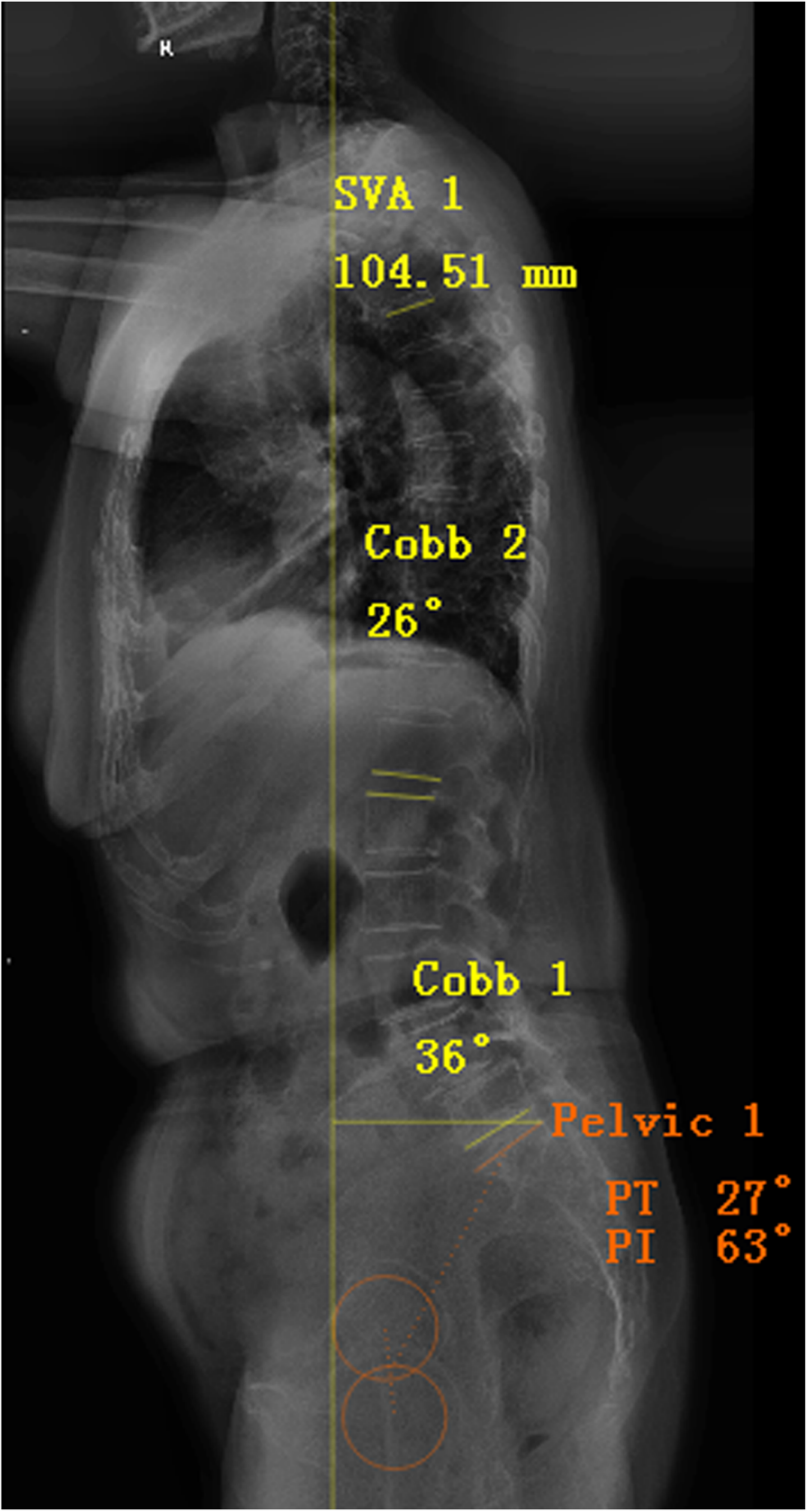
Methods of measurement of spino-pelvic sagittal parameters using Surgimap software

Spino-pelvic sagittal parameters were obtained by the same investigator using Surgimap software [13] for multiple measurements, and the averaged values were used for analyses. The measurements of sagittal parameters were as follows (Fig. 1): (1) SVA was defined as the horizontal distance between the plumb line dropped from the centrum of the seventh cervical vertebra (C7) and the posterior upper corner of the sacral plate. If the SVA fell anterior to the sacrum, it was considered positive, and if it fell behind the sacrum it was considered negative. (2) TK was measured as the angle of intersecting lines drawn from the superior endplate of the fourth thoracic vertebra (T4) and the inferior endplate of the 12th thoracic vertebra (T12). (3) LL was measured as the angle of intersecting lines drawn from the superior endplate of the first lumbar vertebra (L1) and the tangent of the superior endplate of the first sacral vertebra (S1). (4) PI was defined as the angle between the line perpendicular to the S1 superior endplate and the line connecting the midpoint of the S1 superior endplate and the center of the bilateral femoral head. (5) SS was measured between the tangent line to the superior endplate of S1 and the horizontal plane. (6) PT was defined as the angle between the line from the midpoint of the superior endplate of S1 to the center of the bilateral femoral head and the vertical line through the center of the femoral head.

### Statistical analyses

The data of spino-pelvic sagittal parameters were summarized and analyzed by SPSS 19.0 software. Independent sample *t* test was used for normally distributed data to compare differences of sagittal parameters between the observation group and the control group. For data that were not normally distributed, Mann-Whitney *U* test was used. Differences were considered as statistically significant when *p* < 0.05. Pearson correlation test was used to analyze the correlation of the parameters in the two groups. The degree of correlation was expressed by Pearson correlation coefficient “*r*”, and the correlation was considered as statistically significant when *p* < 0.05.

## Results

A total of 167 subjects were enrolled in this study. The observation group had 87 patients (30 males and 57 females) who were admitted to the Orthopedics Department of Beijing Hospital from August 2015 to October 2017. The average age of the group was 75.4 years old (ranging from 70 to 86 years old). The control group had 80 volunteers (28 males and 52 females), with an average age of 74.5 years old (ranging from 70 to 87 years old). No significant difference was found in age between the two groups (Table 1).

**Table 1:** Comparison of age and sagittal parameters between observation group and control group

Parameters	Observation group (*n* = 87)	Control group (*n* = 80)	Statistics	*P*
Sex	30 males; 57 females	28 males; 52 females	–	–
Age (year)	75.45 ± 3.74	74.52 ± 3.64	*u* = 2930.500	> 0.05
SVA (mm)	50.47 ± 36.53	20.13 ± 37.83	*t* = 5.272	< 0.01
TK (°)	29.83 ± 12.30	32.75 ± 8.73	*t* = -1.782	> 0.05
LL (°)	31.98 ± 14.60	43.48 ± 14.09	*u* = 1907.500	< 0.01
PI (°)	48.21 ± 11.84	41.94 ± 8.87	*u* = 2403.500	< 0.01
SS (°)	25.35 ± 11.59	30.19 ± 7.83	*u* = 2347.500	< 0.01
PT (°)	22.24 ± 8.90	11.90 ± 6.66	*u* = 1344.500	< 0.01

The spino-pelvic sagittal parameters were compared between the observation group (*n* = 87) and the control group (*n* = 80) using the data generated by the Surgimap software (Table 1). SVA, PI, and PT of the observation group were significantly higher than those of the control group (*p* < 0.01), while LL and SS were significantly lower in the observation group (*p* < 0.01). No significant difference was found in TK between the two groups.

Correlation analyses of the parameters in the two groups were performed (Table 2). PI was significantly correlated with SS and PT in both the observation group (*p* < 0.01) and the control group (*p* < 0.01), so as the SVA-PI (*p* < 0.05) and SVA-PT (*p* < 0.01). SS-PT was also significantly correlated in the observation group (*p* < 0.01) and in the control group (*p* < 0.05). LL was significantly correlated with all the other parameters in the observation and control groups, including SVA (*p* < 0.01; *p* < 0.01), TK (*p* < 0.01; *p* < 0.01), PI (*p* < 0.01; *p* < 0.01), SS (*p* < 0.01; *p* < 0.01), and PT (*p* < 0.01; *p* < 0.01). SVA-SS (*p* < 0.05), TK-PI (*p* < 0.05) and TK-SS (*p* < 0.01) were significantly correlated in the control group but not in the observation group.

**Table 2:** Correlation of the parameters in the observation group and in the control groups

Parameters	Observation group		Control group	
	*r*	*p*	*r*	*p*
SVA-TK	− 0.164	> 0.05	− 0.035	> 0.05
SVA-LL	− 0.308	< 0.01	− 0.442	< 0.01
SVA-PI	0.250	< 0.05	0.238	< 0.05
SVA-SS	− 0.091	> 0.05	− 0.266	< 0.05
SVA-PT	0.454	< 0.01	0.652	< 0.01
TK-LL	0.501	< 0.01	0.609	< 0.01
TK-PI	0.119	> 0.05	0.249	< 0.05
TK-SS	0.070	> 0.05	0.423	< 0.01
TK-PT	0.064	> 0.05	− 0.163	> 0.05
LL-PI	0.577	< 0.01	0.488	< 0.01
LL-SS	0.812	< 0.01	0.907	< 0.01
LL-PT	− 0.289	< 0.01	− 0.430	< 0.01
PI-SS	0.706	< 0.01	0.683	< 0.01
PI-PT	0.408	< 0.01	0.498	< 0.01
SS-PT	− 0.357	< 0.01	− 0.280	< 0.05

## Discussion

Sagittal parameters of elderly patients have their own characteristics compared to young people. The present study enrolled 80 volunteers at age ≥ 70 years old as the control group, and their sagittal parameters were found to have a large range of variation. Compared to the previously reported sagittal parameters of normal young people [10, 14], there was a clear difference. The surgical treatment of elderly lumbar degenerative disease requires a restoration of spinal morphology and reconstruction of the spino-pelvic sagittal balance, especially the lumbar lordosis. When predicting the required correction of LL, normal values of spino-pelvic sagittal parameters of the same age group may be more appropriate than using the values established from young people.

Previous studies showed that elderly patients were mostly in the state of sagittal imbalance, with the trunk center of gravity moving forward, the integral sagittal alignment becoming straight, and the pelvic posterior tilt increasing. A retrospective study by Rajnics et al. showed that patients with lumbar disc herniation had more straight sagittal curve compared with healthy adults, with SVA, PT increased, and SS and LL decreased; however, there was no significant difference in PI and TK [15]. Other studies also found that patients with lumbar degenerative disease had decreased SS, LL, and TK and increased SVA and PT. PI was normal in most of the cases, and the reduction became significant only in patients < 45 years old [16, 17]. In a study comparing the PI values of 200 patients with chronic low back pain and 89 normal adults, no significant difference in the PI values between patients < 60 years old and normal subjects of the same age group was found [18]. However, for patients > 60 years old, PI was significantly greater than that of the normal subjects. In the present study, SVA and PT of the observation group were significantly higher than that of the control group, while LL and SS of the observation group were significantly lower than that of the control group, consistent with the previous studies. The PI values of the observation group were significantly higher than that of the control group, which is consistent with the above findings. The results indicated that elderly patients with lumbar degenerative disease are mostly in the state of sagittal imbalance, which leads to loss of lumbar lordosis, trunk center of gravity moving forward, and standing instability.

No significant difference in TK was found in this study. This may be due to the lower thoracic activity in elderly patients; therefore, this compensatory mechanism is often not obvious.

It is believed that pelvic parameters correlate with each other and have mutual influence on spinal parameters [7, 19–21]. Our data showed that LL was the only parameter that correlated with all the other parameters, suggesting LL was a core parameter of spino-pelvic sagittal balance. On the other hand, PI was correlated with SS and PT in both the observation and control groups, which are in consistent with previous findings. In addition, the values of LL, PT, and SVA were intercorrelated, indicating the pelvic morphology and spinal sagittal sequence are closely related. The pelvis compensates for the sagittal imbalance of the spine by two mechanisms: one is to change the curvature of the lower lumbar by adjusting the direction of the sacral plateau, and the second is to change the center of gravity of the trunk by rotating the pelvis.

The spine and pelvis coordinate well with each other to maintain the balance in healthy individuals. In this study, SVA-SS, TK-PI, and TK-SS were significantly correlated in the control group, but such correlations were not found in the observation group, indicating that coordination of the spine and pelvis were reduced in elderly patients with lumbar degenerative disease.

There are limitations of the current study: (1) all the subjects were from the single institute, and a larger number of samples from multi-center will be needed for validation; (2) characteristics such as height and weight were not taken into account; (3) elderly lumber degenerative disease include different subtypes, and a subtype analysis with a larger sample size is beneficial; and (4) the limitation of adequacy to compare the control (volunteer) with outpatients should be realized.

Sagittal parameters of elderly patients with a degenerative spine have their own characteristics compared to young people. The data collected in the control group of this study could provide a reference for planning of the surgical treatment of the spinal diseases in elderly patients. Our data showed that pelvic parameters were significantly correlated with each other, which may affect the sagittal curve of the spine. LL was significantly correlated with all the other sagittal parameters, suggesting its core role in spino-pelvic sagittal balance. With further validation of the findings, the information will be helpful for management of elderly patients with degenerative spine.

## Abbreviations

LL: Lumbar lordosis
PI: Pelvic incidence
PT: Pelvic tilt
SS: Sacral slope
SVA: Sagittal vertical axis
TK: Thoracic kyphosis
